# Genome-wide SNP analysis provides insights into the XX/XY sex-determination system in silver barb (*Barbonymus gonionotus*)

**DOI:** 10.5808/gi.23075

**Published:** 2023-12-29

**Authors:** Visarut Chailertrit, Thitipong Panthum, Lalida Kongkaew, Piangjai Chalermwong, Worapong Singchat, Syed Farhan Ahmad, Ekaphan Kraichak, Narongrit Muangmai, Prateep Duengkae, Surin Peyachoknagul, Kyudong Han, Kornsorn Srikulnath

**Affiliations:** 1 Animal Genomics and Bioresource Research Unit (AGB Research Unit), Faculty of Science, Kasetsart University, Bangkok 10900, Thailand; 2 Pathum Thani Aquatic Animal Genetics Research and Development Center, Aquatic Animal Genetics Research and Development Division, Department of Fisheries, Pathum Thani 12120, Thailand; 3 Laboratory of Animal Cytogenetics and Comparative Genomics (ACCG), Department of Genetics, Faculty of Science, Kasetsart University, Bangkok 10900, Thailand; 4 Special Research Unit for Wildlife Genomics (SRUWG), Department of Forest Biology, Faculty of Forestry, Kasetsart University, Bangkok 10900, Thailand; 5 Sciences for Industry, Faculty of Science, Kasetsart University, Bangkok 10900, Thailand; 6 Department of Botany, Kasetsart University, Bangkok 10900, Thailand; 7 Department of Fishery Biology, Faculty of Fisheries, Kasetsart University, Bangkok 10900, Thailand; 8 Department of Microbiology, Dankook University, Cheonan 31116, Korea; 9 Bio-Medical Engineering Core Facility Research Center, Dankook University, Cheonan 31116, Korea; 10 Center for Advanced Studies in Tropical Natural Resources (CASTNAR), National Research University-Kasetsart University (NRU-KU), Kasetsart University, Bangkok 10900, Thailand

**Keywords:** sex determination, silver barb, single-nucleotide polymorphism, Y chromosome

## Abstract

Silver barb (*Barbonymus gonionotus*) is among the most economically important freshwater fish species in Thailand. It ranks fourth in economic value and third in production weight for fisheries and culture in Thailand. An XX/XY sex-determination system based on gynogenesis was previously reported for this fish. In this study, the molecular basis underlying the sex-determination system was further investigated. Genome-wide single-nucleotide polymorphism data were generated for 32 captive-bred silver barb individuals, previously scored by phenotypic sex, to identify sex-linked regions associated with sex determination. Sixty-three male-linked loci, indicating putative XY chromosomes, were identified. Male-specific loci were not observed, which indicates that the putative Y chromosome is young and the sex determination region is cryptic. A homology search revealed that most male-linked loci were homologous to the Mariner/Tc1 and Gypsy transposable elements and are probably the remnants of an initial accumulation of repeats on the Y chromosome from the early stages of sex chromosome differentiation. This research provides convincing insights into the mechanism of sex determination and reveals the potential sex determination regions in silver barb. The study provides the basic data necessary for increasing the commercial value of silver barbs through genetic improvements.

## Introduction

With increasing world population, there is a need to improve food security, which calls for targeted actions to achieve zero hunger under the Sustainable Development Goals adopted by the United Nations in 2015. Currently, fish is the primary source of animal protein for one billion people. Global fish production was 214 million tons in 2020; 157.4 million tons was used for human consumption, and an increasing dependence on production from capture fisheries and aquaculture was reported [1]. Declining fish stocks in oceans, rivers, and lakes pose a threat to people who are dependent on catching fish for their sustenance or are employed in the fishery industry. The efficiency of the aquaculture sector, which contributes significantly to global food production, can be improved through genetic enhancement [2]. Silver barb (*Barbonymus gonionotus*, Bleeker, 1849) is an important food fish in Southeast Asia with high protein and a delicious taste and is a promising target for aquaculture [3]. Ranked fourth in economic value and third in production weight for both fisheries and culture, the silver barb is among the economically significant freshwater species in Thailand [4]. It can withstand high stocking densities and attains a marketable size within 3–4 months [5]. Because female silver barbs grow significantly faster than males, production of all-female silver barbs has substantial economic implications for aquaculture [6].

The ability to control sex and breeding aids the production of large stocks in hatcheries, particularly in the reliable production of specific family combinations for selective breeding [7]. Without this ability, farmers have little control over breeding for genetic improvements. The control of sex and reproduction has been the primary enabler in large-scale global industrial aquaculture production. Currently, all-female silver barb offspring can be produced by gynogenesis, whereby the genome of the embryo has exclusively female origin following embryogenesis simulation by a male gamete [8,9]. The identification of monosex female offspring through gynogenesis led to the hypothesis that the sex-determination system (SDS) in the silver barb is XX/XY. However, the molecular basis for this SDS is poorly understood, and no heteromorphic chromosomes have been identified between females and males [10]. An understanding of the SDS in silver barb is, therefore, an important baseline for future research on evolutionary biology, sex development, and genetic improvement for aquaculture.

Advanced high-throughput molecular methods, in combination with next-generation sequencing technologies, such as restriction site-associated DNA sequencing (RAD-seq) comprising double-digest RAD-seq (ddRADseq) and 2b-RAD sequencing, and diversity arrays technology sequencing (DArTseq), have been applied to identify genotypes [11-14]. These methods are effective in identifying sex-linked markers in non-model species using single-nucleotide polymorphism (SNP) loci. Remarkably, DArTseq markers can reveal sex-associated loci, thereby, facilitating the identification of sex-determining regions in cryptic sex chromosomes of non-model species [15-18]. We investigated the SDS of silver barb employing a genome-wide SNP approach using DArTseq from pre-sexed (based on their phenotype) captive-bred individuals. The genetic understanding of the SDS of this cultured species should assist in aquaculture development.

## Methods

### Specimen selection and DNA extraction

A full-sib family of silver barb was artificially fertilized and cultured at the Pathum Thani Aquatic Animal Genetics Research and Development Center, Aquatic Animal Genetics Research and Development Division, Department of Fisheries, Ministry of Agriculture and Cooperatives, Thailand. A total of 32 samples (16 males and 16 females) of 4-month-old adults with standard weights of 10–12 g and lengths of 10–15 cm were euthanized and preserved in 95% ethanol. Whole genomic DNA was extracted following the standard salting-out protocol, with slight modifications for different tissues [19]. High-molecular-weight DNA samples were stored at −20°C until required for the construction of DArTseq library, as described previously [20]. All experimental procedures were approved (approval No. ACKU63-SCI-007) by the Animal Experiment Committee of Kasetsart University and conducted in accordance with the Regulations on Animal Experiments at Kasetsart University.

### DArT sequencing and genotyping

The DArTseq methodology for sequencing and genotyping by SNP loci was applied following the protocol described by Jaccoud et al. (2001) [20]. Multiple loci were genotyped using DArTseq (Diversity Arrays Technology Pty Ltd., Canberra, Australia) to identify the SNP loci and silico DArT markers (also called presence/absence [PA] markers, as any variability in the SNP loci generates PA polymorphisms in restriction sites) were used. The data were used to determine the sex-candidate loci in both male and female individuals. Approximately 100 ng of DNA was collected from each specimen to develop the DArTseq arrays. The DNA samples were subjected to digestion and ligation [17,21]. The outputs generated by DArTsoft14 were filtered according to predefined criteria, including reproducibility values (>3.5), average sequence count (sequencing depth > 5), balance of SNP allele counts (>0.9), and call rate (>0.8), as previously described [17]. Sex-specific and sex-linked loci were identified using SNP and PA marker analyses. For an XX/XY SDS, male-specific data sets were created, with loci sequenced at various percentages (70%, 80%, 90%, and 100%). The loci that passed the 100% filtering were designated as sex-specific, whereas those within the 70%–90% threshold were classified as sex-linked. An opposite and similar approach was used to target loci based on the ZZ/ZW system. The Hamming distance was calculated to determine the number of combined loci between male and female individuals to identify the pairwise differences in SNP and PA loci using the “rdist” function in R version 3.5.1 [22]. The Hamming distance represents the number of pairwise differences between all individuals across all loci. The Cochran-Armitage trend test (CATT) was used to examine the genetic association between each locus and phenotypic sex in the SNP and PA loci using the “catt” function in the HapEstXXR package of R version 3.5.1. The CATT results were consistent with those of a chi-square test used to examine whether the observed genotype proportions conformed to the expected values. The polymorphic information content (PIC), which is an index for evaluating the informativeness of SNP and PA loci, was calculated for each locus and ranged from 0 (fixation of one allele) to 0.5% (the frequencies of both alleles were equal) [22-24]. The probability of the sex-linked loci showing random associations with sex when using a small sample size was estimated using the formula P_i_ = 0.5^n^, where P is the probability for a given locus, i is sex-linked, 0.5 is the probability that either a female is homozygous or a male is heterozygous at a given locus, and n is the number of individuals sequenced at the locus [24]. The full dataset and metadata of this publication are available from the Dryad Digital Repository. Dataset, https://datadryad.org/stash/share/P6fDtif_Ig3ZLeYfYCc98BUadnpzHBZXkG8wYoNL-w8 (https://doi.org/10.5061/dryad.hhmgqnkhn.)

### Comparison of potential sex-linked loci

Significant differences among the three groups of sex-linked loci (90:10, 80:20, and 70:30) were analyzed using the chi-squared test and Kruskal-Wallis test for PA loci and the Nemenyi test for SNP loci, using the “PMCMR” package in R [22]. The mean heterozygosity and standard deviation of the loci were analyzed. All candidate loci were plotted for each individual using the “glPlot” function in the “dartR” package in R [22]. A visual representation of the results was obtained through a principal coordinate analysis using all groups of sex-linked loci [22,24].

### *In silico* chromosome mapping

Owing to the unavailability of a chromosome-level assembly for the silver barb, the sex-candidate loci were aligned to the chromosome-level assembly of the common barbel (*Barbus barbus*) (accession Nos. OW387152–OW387166 and OW387168–OW387202) using NCBI-BLASTn with default parameters [25]. The output-mapped file was filtered with the most significant hits (identity: >95%; alignment length: >65 bp) and then parsed using custom Geneious Prime 2023.1.2 (Biomatters, Auckland, New Zealand; https://www.geneious.com) to generate a file format for visualization of the chromosome map.

### Homology search

The sex-candidate loci showing a statistically significant association with the known sex phenotype were subjected to a BLAST search using the National Center for Biotechnology Information (NCBI) database. Homologies between the sex-specific/linked SNP/PA loci and the reference genomes of other teleosts, including Japanese rice fish (*Oryzias latipes*, Temminck and Schlegel, 1850; accession No. GCF_002234675.1) [26], zebrafish (*Danio rerio*, Hamilton, 1822; accession no. GCA_000002035.4) [27], Japanese pufferfish (*Takifugu rubripe*, Temminck and Schlegel, 1850; accession No. GCA_901000725.2) [28], and chicken (*Gallus gallus*, Linnaeus 1758; accession No. AADN00000000.5; International Chicken Genome Sequencing Consortium 2004) were investigated. The NCBI database and RepBase version 19.11 (Genetic Information Research Institute, http://www.girinst.org/repbase/) were used to search for homologies of all loci using the BLASTn program [29]. RepBase is a specialized database with repeated or other significant sequences and only reports results with E-values < 0.005 and a query coverage with >55% similarity [24].

### Functional annotation and gene ontology of the silver barb

Functional annotation was performed to understand the biological functions of the sex-specific/linked SNP loci. BLASTn was performed with all candidate loci against the reference annotation consisting of the gene dataset of common barbel [30]. A reference gene dataset was retrieved from the Ensembl database using the Biomart package (https://www.ensembl.org/index.html). BLASTn results were generated as a tabular formatted output file, and only significant hits (identity >95% and alignment length >65 bp) were retained. All gene sequences from the reference dataset that corresponded to the region with significant hits were extracted and mapped against the proteome dataset (including total annotated proteins). The proteome dataset was downloaded from UniProtKB/Swiss-Prot [31]. UniProtKB is a protein database that provides comprehensive and reliable information on protein functions through accurate, consistent, and detailed annotations. Functional annotations and Gene Ontology (GO) enrichment analyses were also performed on the filtered gene hits using ShinyGO (0.77) implemented in the R/Bioconductor packages. The best-matching species genome was used as a reference in the analysis, with standard settings that included a 0.05 false discovery rate (fold enrichment) p-value threshold [32]. Associated GO terms describing biological processes (BPs), molecular functions (MFs), and cellular components (CCs) were detected by processing the matching transcripts. GO categories were identified using UniProtKB, the Gramene Protein Database (GR_protein), and the Protein Data Bank (PDB).

## Results

### Determination of the sex system and identification of sex-candidate loci in the silver barb

**Fig. 1.**
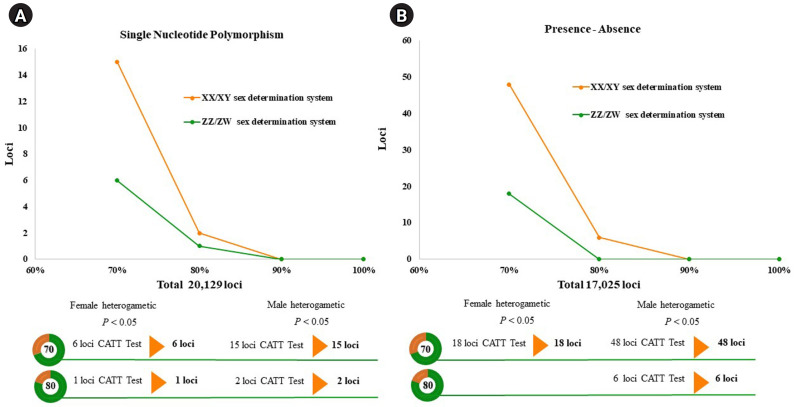
Line charts displaying the threshold of the single-nucleotide polymorphism and the presence/absence (PA) loci in the silver barb (*Barbonymus gonionotus*, Bleeker, 1849). (A) Single-nucleotide polymorphism loci filtered with 30:70 and 20:80 (female:male) ratios. (B) PA loci filtered using the 30:70 and 20:80 (female:male) ratios. CATT, Cochran-Armitage trend test.

**Fig. 2.**
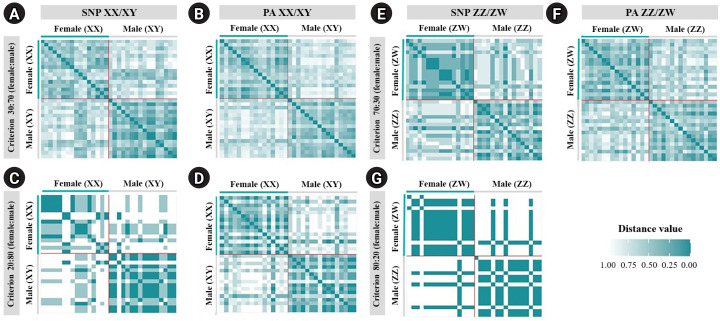
Hamming distances of the male and female silver barb (*Barbonymus gonionotus*, Bleeker, 1849) determined using single-nucleotide polymorphism (SNP) and presence/absence (PA) loci. (A) SNP loci filtered using the 30:70 (female:male) criterion. (B) PA loci filtered using the 30:70 (female:male) criterion. (C) SNP loci filtered using the 20:80 (female:male) criterion. (D) PA loci filtered using the 20:80 (female:male) criterion for the XX/XY system. (E) SNP loci filtered using the 70:30 (female:male) criterion. (F) PA loci filtered using the 70:30 (female:male) criterion. (G) SNP loci filtered using the 80:20 (female:male) criterion for the ZZ/ZW system.

A total of 20,129 SNP and 17,025 PA loci were examined in 32 individuals, including 16 males and 16 females. The PIC values for SNP loci ranged from 0.03 to 0.50, with an average of 0.22, while those for PA loci ranged from 0.06 to 0.50, with an average of 0.30. These results indicate that the overall distribution of PIC values was asymmetrical and skewed toward higher values. The number of filtered SNPs and PAs was then compared between male and female groups after filtering. For the XX/XY type, applying a 30:70 (female:male) criterion resulted in 15 SNP and 48 PA loci. (Fig. 1A and 1B). These loci were significantly associated with the phenotype based on CATT analysis (*χ*^2^ = 4.62–16.35, p < 0.05). The Hamming distance within sexes was 0.403 ± 0.018 in males and 0.525 ± 0.018 in females for SNP loci, and 0.463 ± 0.014 in males and 0.487 ± 0.016 in females for PA loci. The between-sex distances were 0.688 ± 0.012 and 0.694 ± 0.009 for the SNP and PA loci, respectively (Fig. 2A and 2B). Additionally, filtering using the 20:80 (female:male) criterion revealed 2 SNP loci and 6 PA loci (Fig. 1A and 1B). These loci were significantly associated with the phenotype based on CATT analysis (*χ*^2^ = 10.29–16.35, p < 0.05). The Hamming distance within sexes was 0.342 ± 0.034 in males and 0.575 ± 0.036 in females for SNP loci and was 0.412 ± 0.026 in males and 0.462 ± 0.026 in females for PA loci. The between-sex distances were 0.807 ± 0.018 and 0.769 ± 0.015 for the SNP and PA loci, respectively (Fig. 2C and 2D). However, no sex-candidate loci were identified when using the 10:90 or 0:100 (female:male) criteria (Fig. 1A and 1B).

**Fig. 3.**
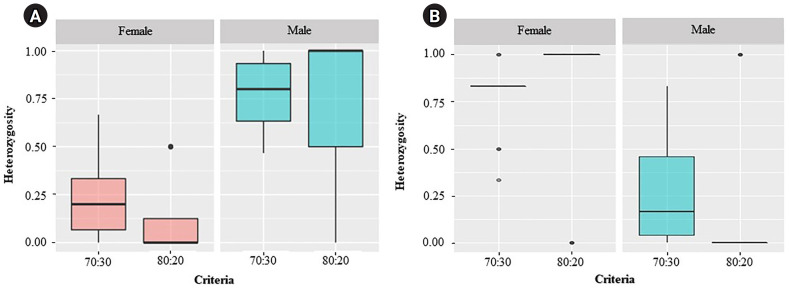
Kruskal-Wallis analysis showed no significant difference in the percentages of heterozygosity for single-nucleotide polymorphism in males (H = 1.82, p = 0.177) and females (H = 4.74, p = 0.0295) with the XX/XY sex-determination system (A), and in males (H = 0, p = 1) and females (H = 0, p = 1) with the ZZ/ZW sex-determination system (B). Bullets indicate the outside median ± interquartile range (Q3–Q1).

**Fig. 4.**
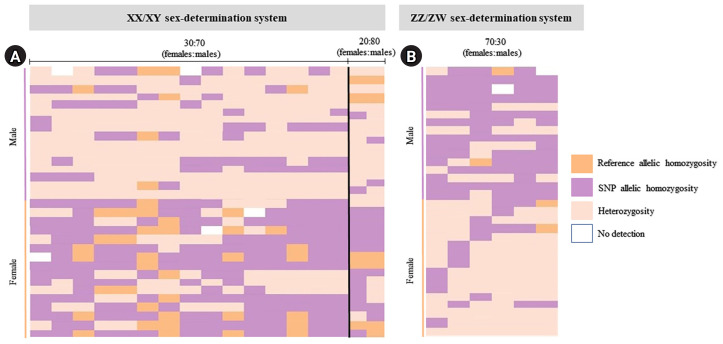
Index of 32 moderately sex-linked loci. Loci with the 30:70 (female:male) criteria (the XX/XY sex-determination system) (A) and loci with the 30:70 (female:male) criteria (the ZZ/ZW sex-determination system) (B) created using the “glPlot” function in the R package, “*dartR*.” Orange indicates homozygosity to the reference allele, pink indicates heterozygosity, and purple indicates homozygosity to the allele containing the alternate single-nucleotide polymorphism (SNP).

For the ZZ/ZW type, filtering using the 70:30 (female:male) criterion revealed 6 SNP and 18 PA loci (Fig. 1A and 1B). These loci were significantly associated with the phenotype based on CATT analysis (*χ*^2^ = 5.81–15.24, p < 0.05). The Hamming distance within sexes was 0.449 ± 0.022 in males and 0.365 ± 0.021 in females for SNP loci and was 0.474 ± 0.017 in males and 0.453 ± 0.017 in females for PA loci. The between-sex distances were 0.671 ± 0.014 and 0.668 ± 0.011 for the SNP and PA loci, respectively (Fig. 2E and 2F). Moreover, filtering using the 80:20 (female:male) criterion resulted in only one SNP locus and no PA loci (Fig. 1A and 1B). This locus was significantly associated with the phenotype based on CATT analysis (*χ*^2^ = 15.24, p < 0.05). The Hamming distance within sexes was 0.425 ± 0.045 in males, 0.342 ± 0.044 in females, and 0.801 ± 0.025 between the sexes for the SNP loci (Fig. 2G). However, no sex-specific SNP/PA loci were found with 90:10 or 100:0 (female:male) criterion (Fig. 1A and 1B). The The Kruskal-Wallis test indicated no significant differences in heterozygosity percentages for SNPs in males (*H* = 1.82, p = 0.177) or females (*H* = 4.74, p = 0.0295) with XX/XY sex-determination. Similarly, for ZZ/ZW sex determination, no significant differences were observed in males (*H* = 0, p = 1) and females (*H* = 0, p = 1). (Fig. 3). A principal coordinate analysis plot demonstrated a more similar grouping between the sexes (Fig. 4).

### Random sex-linkage estimation

A range of sample sizes and loci were collected from 32 individuals of the silver barb to minimize the probability of selecting less than one spurious sex-linked marker. For the 32 specimens, the P_i_ (i.e., probability of a single locus exhibiting a sex-linked pattern by chance) was 2.33 × 10^-10^ based on 37,154 loci (including SNP and PA loci). The expected sex linkage was 8.65 × 10^-6^. The number of random sex-linked markers in the silver barb was lower than the expected values.

### Chromosome localization of sex-linked loci based on *in silico* mapping

*In silico* chromosome mapping of all sex-linked loci of the silver barb onto the chromosome-level assembly of common barbel (accession Nos. OW387152–OW387166 and OW387168–OW387202) revealed that 32 of 63 sex-link loci of the silver barb were localized to 22 of 50 chromosomes of the common barbel. Four loci were localized to chromosome 11, whereas chromosome 2, 7, 8, 25, 35, 41, and 44 were mapped with two loci in each. Only one locus each mapped onto chromosome 3, 4 10, 14, 17, 20, 21, 29, 30, 32, 42, 43, 48, and 50 (Supplementary Fig. 1).

### Homology of putative sex-linked loci

Sex-linked loci in male silver barb shared a sequence homology with the Japanese rice fish, zebrafish, Japanese pufferfish, and chicken genomes (Supplementary Table 1). In the global BLAST analyses using the NCBI databases, six of the 63 male-linked loci were homologous with putative genes: *HoxAa* (homeobox) (E-value 4.00 × 10^-3^, 59% similarity), *TEF* (transcriptional enhancer factor) (E-value 8.00 × 10^-10^, 66% similarity), *APOL3* (apolipoprotein L3) (E-value 5.00 × 10^-8^, 97% similarity), *prkra* (protein activator of interferon-induced protein kinase EIF2AK2) (E-value 1.00 × 10^-10^, and 97% similarity), *snrnp70* (small nuclear ribonucleoprotein U1 subunit 70) (E-value 0.045, 66% similarity), and *Nek4* (serine/threonine-protein kinase) (E-value 8.00 × 10^-6^, 98% similarity) (Table 1). Not all the loci were included in the sex developmental pathway. Additionally, 16 male-linked loci showed partial homology with transposable elements (TEs), mostly Mariner/Tc1 and Gypsy (Table 2).

### Functional classification and enrichment analysis of the silver barb loci

Specific SNP loci in the silver barb were subjected to GO enrichment analyses. The GO-enriched categories of BP terms were mainly involved in the regulation of transport and regulation of vesicle-mediated transport, and the MF terms were mostly related with lipid and phosphatidylinositol binding, and the CC terms were mostly connected with plasma membrane region, cell leading edge, and lamellipodium (Supplementary Fig. 2).

## Discussion

Latest technologies in aquaculture have been developed from extensive to semi-intensive culture systems on a commercial scale; however, further research is required to enhance production and stock quality while improving fish health management [2]. One major research area is the chromosome-level manipulation for improving aquacultural traits [33-35]. Successful chromosome manipulation in fish species with known SDS has enabled controlled breeding and size dimorphism for efficient husbandry and production management [36]. Chromosome manipulation is crucial for gynogenesis and enables efficient production and cloning of all-female individuals in fish species like the silver barb. Gynogenesis also serves as a valuable tool for investigating SDS in aquaculture research [36]. Male heterogamety was observed in silver barb, with 63 male-linked loci exhibiting genome-wide SNP patterns. This suggests an XX/XY SDS in the silver barb, with all male-linked loci located on a putative Y chromosome. Four of the 63 male-linked loci were mapped onto chromosome 11 of the common barbel, and several loci were localized to different chromosomes. This suggests that many male-linked loci were false-positive loci. By contrast, large chromosomal rearrangements were often observed in teleosts, even at the same genus level [37-39]. Silver barb and common barbel are not at the same genus level, and intra- and interchromosomal rearrangements might result in chromosomal linkage reshuffling, whereas about half of the male-linked loci of the silver barb were informatically mapped onto common barbell chromosomes. All male-linked loci might be retained on the same linkage in the silver barb. However, whether the locations of large genomic regions containing the X- and Y-specific fragments are associated with sex chromosome differentiation and sex-determining regions remains unclear. A major challenge in mapping these loci on sex chromosomes is their short sequence generated using DArTseq and diverse genetic backgrounds that give many false-positive signals [46]. The probability of spurious sex linkage for a single locus in the full data set of 37,154 loci (including SNP and PA loci) was 2.33 × 10^-10^, whereas the expected level of sex linkage was estimated to be 8.65 × 10^-6^. The observed male-linked loci in the silver barb exceeded the expected value in this study.

Out of the 63 male-linked loci, six shared partial homology with functional genes. Interestingly, one locus (PA57951108) was homologous with sex chromosomal linkage in amniotes. This result was similar to the comparative homology of sex-specific and linked loci in several teleosts, such as bighead catfish (*Clarias macrocephalus*), snakeskin gourami (*Trichopodus pectoralis*), Siamese fighting fish (*Betta splendens*), and other amniotes, and indicates the possibility of a super-sex chromosome in ancestral amniotes [15,17,18]. Convergent evolution is assumed to be the driving force that causes divergence of sex chromosomes among phylogenetically distant or closely related taxa [47,48]. We also detected certain sex-linked loci that showed significantly similar retroelements, such as Mariner/Tc1 and Gypsy, which are frequently distributed on sex chromosomes in Japanese rice fish (*Oryzias latipes*, Temminck and Schlegel, 1850) platyfish (*Xiphophorus maculatus*, Günther, 1866), pufferfish (*Takifugu * rubripes, Temminck and Schlegel, 1850), and tilapia (*Oreochromis niloticus*, Linnaeus, 1758) [49-51]. Chromosomal rearrangements mediated by TEs can induce sex chromosome differentiation and repositioning of heterochromatin. Sex chromosomes of different species were enriched in TEs, indicating that the possible initial accumulation of TEs in the Y chromosome during the early stage of sex chromosome differentiation in the silver barb [49,52-55].

Only one female-linked locus passed the CATT test, possibly because of partial recombination in the silver barb. Both male- and female-linked loci were occasionally observed in the same silver barb individual but also occurred in different linkage groups (Supplementary Table 1). There are two possible explanations for the coexistence of both male- and female-linked loci: (1) frequent recombination within the regions of homomorphic sex chromosomes [56], or (2) putative interactions with other minor genes from male-linked loci in the same linkage group with environmental factors such as temperature [57]. This might locate all male-linked loci in the same linkage group, with a genetics-based sex-determining mechanism involving a major sex-determining region. Other minor genetic or environmental factors cannot be disregarded [57]. The SDS in teleosts represent a highly dynamic and plastic phenomenon that triggers gonadal development [58]. This high-level dynamism is very important for sexual reproduction and survival of a species but sex-determination mechanisms are extremely complex and highly variable [17]. Genomic resources and tools for the silver barb have improved our understanding of sex determination in this fish. Further investigation is needed to explore sex linkage variability in different populations. Challenges include short read lengths in genotyping techniques and random biological variation, which may result in the identification of sex-linked loci outside of the sex-determination regions or even on autosomes, especially when the sample sizes are small [59].

The XX/XY SDS has implications for sex-controlled breeding in silver barb aquaculture. Various techniques, such as exogenous hormone treatment, chromosome ploidy manipulation, gynogenesis, molecular tools, and hybridization, can be employed to produce monosex populations. These methods offer advantages, including the production of larger silver barb, which commands higher prices. Monosex populations are also associated with reduced variability compared with mixed sex groups [8]. The ability to control sex and breeding is pivotal for hatcheries to produce large stocks, particularly reliable specific family combinations through selective breeding. Sex-linked genetic markers and marker-assisted breeding techniques play a vital role in selective breeding. They enable the production of single-sex cohorts in species without visible sexual dimorphism until sexual maturity [60]. In this study, we encountered several challenges and none of the 63 male-candidate loci independently discovered in the silver barb were successfully validated. Few female individuals showed a nonspecific banding pattern, possibly as a result of an unstable primer binding site. Therefore, this method was not effective in confirming the sex-linked markers (data not shown). Genotyping using sequencing technologies, such as DArTseq or RAD-seq, was also not appropriate for PCR-based validation [46,50,61]. Failure of PCR can be due to conserved regions near sex-specific restriction sites in both sexes [62]. Developing alternative PCR-based genotyping tools is necessary to accurately assess and compare sex-linked loci within populations. Methods, such as polymerase chain reaction-restriction fragment length polymorphism or melting curve analysis, offering more sensitive detection, may be suitable for sex validation [16,46,50,61,63]. Extensive analysis of large sample sizes from diverse population groups, together with the development of optimized techniques, can further validate sex-linked markers in the silver barb.

The findings of this study together with previous gynogenesis research [8,9] suggest the existence of XX/XY SDS in the silver barb. Data from a variety of trials and other sources indicate that sexual dimorphism increases with size. Female silver barb grows significantly faster than males, providing improved production with higher yields. The maximum gain from monosex culture would be expected in systems where individuals are grown to a large size and/or to maturity if the target market comprises ovary consumers. However, further elucidation of sex-determining genes and sex chromosome linkage groups is required before the silver barb biological constraints are fully understand and before we have a baseline for genetic manipulation in aquaculture. This research ushers in a new era for studying the genetic basis of sexual dimorphism using biotechnological manipulation for sex-controlled breeding.

## Figures and Tables

**Table 1.** Gene function and pathway analyses for presence/absence loci in the silver barb (*Barbonymus gonionotus*, Bleeker, 1849) using a BLAST homology search of the genomes of Japanese rice fish (*Oryzias latipes*, Temminck and Schlegel, 1850), zebrafish (*Danio rerio*, Hamilton, 1822), Japanese pufferfish (*Takifugu rubripes*, Temminck and Schlegel, 1850), Barramundi (*Lates calcarifer*, Bloch, 1790), and chicken (*Gallus gallus*, Linnaeus, 1758) (30:70, female:male; XX/XY sex-determination type)

Locus ID	Gene	Product	Function	Pathway	Reference
PA57951923	*HoxAa*	Homeobox (HoxAa)	Transcription factor, morphogenesis	Neural pathway	Lambert et al. (2012) [40]
PA57973663	*TEF*	Transcriptional enhancer factor	Governs cell growth, proliferation, and apoptosis	Hippo signaling pathway	Zhang et al. (2008) [41]
PA57953141	*APOL3*	Apolipoprotein L3	Innate immunity genes	Secretory pathway	Pant et al. (2021) [42]
PA57952726	*PRKRA*	Protein activator of interferon induced protein kinase EIF2AK2	Protein PACT	Neural pathway	Vaughn et al. (2015) [43]
PA57952528	*SNRNP70*	Small nuclear ribonucleoprotein U1 subunit 70	Cell adhesion	Cadherin mediated pathway	Nakaya (2020) [44]
PA57953141	*NEK4*	Serine/threonine-protein kinase	Control cell growth	Regulatory pathways	Motose et al. (2012) [4]

**Table 2.** Repeat searches for single-nucleotide polymorphism and restriction fragment presence/absence male-linked loci in the silver barb (*Barbonymus gonionotus*, Bleeker, 1849)

Repeat	Type	Male-linked loci	
		SNP loci	PA loci
DNA transposon	Harbinger	-	1
	Kolobok	-	3
	Mariner/Tc1	-	6
	hAT	-	-
Non-LTR retrotransposon	L1	-	-
LTR retrotransposon	Gypsy	4	2
	Copia	-	-

SNP, single-nucleotide polymorphism; PA, presence/absence; LTR, long terminal repeats.

## Supplementary Materials

Supplementary Table 1. Quality of the sequencing data including Call Rate, AvgReadDepth (Average read depth), and RepAvg (Repeatability averaged) and Chromosomal locations of other vertebrates using the homologous sequences to single-nucleotide polymorphisms and restriction fragment presence/absence loci in silver barb (*Barbonymus gonionotus*, Bleeker, 1849) from a BLAST homology search of the genomes of Japanese rice fish (*Oryzias latipes*, Temminck and Schlegel, 1850), zebrafish (*Danio rerio*, Hamilton, 1822), Japanese pufferfish (*Takifugu rubripes*, Temminck and Schlegel, 1850), and chicken (*Gallus gallus*, Linnaeus, 1758) (30:70, female: male; XX/XY sex-determination type)

Supplementary Fig. 1. *In silico* chromosome mapping showing the distribution of sex-linked loci of the silver barb to the chromosome-level assembly of the common barbel.

Supplementary Fig. 2. Gene ontology (GO) functional classification of specific loci of the silver barb using Blast2GO. Histograms of the frequency of transcripts annotated to specific GO categories, viz., biological process, molecular functions, and cellular components, are represented using orange, blue, and green bars, respectively.
