# Supplementary material for: Genome-wide SNP analysis provides insights into the XX/XY sex-determination system in silver barb (Barbonymus gonionotus)
Source: Genomics Inform. 2023 Dec 29;21(4):e47. doi: 10.5808/gi.23075 (PMC10788355; doi:10.5808/gi.23075)
Supplement: Supplementary Table 1. — Quality of the sequencing data including Call Rate, AvgReadDepth (Average read depth), and RepAvg (Repeatability averaged) and Chromosomal locations of other vertebrates using the homologous sequences to single-nucleotide polymorphisms and restriction fragment presence/absence loci in silver barb (Barbonymus gonionotus, Bleeker, 1849) from a BLAST homology search of the genomes of Japanese rice fish (Oryzias latipes, Temminck and Schlegel, 1850), zebrafish (Danio rerio, Hamilton, 1822), Japanese pufferfish (Takifugu rubripes, Temminck and Schlegel, 1850), and chicken (Gallus gallus, Linnaeus, 1758) (30:70, female: male; XX/XY sex-determination type) [file gi-23075-Supplementary-Table-1.pdf]

Supplementary Table 1. Quality of the sequencing data including Call Rate, AvgReadDepth (Average read depth), and RepAvg (Repeatability averaged) and Chromosomal locations of other vertebrates using the homologous sequences to single-nucleotide polymorphisms and restriction fragment presence/absence loci in silver barb (*Barbonymus gonionotus*, Bleeker, 1849) from a BLAST homology search of the genomes of Japanese rice fish (*Oryzias latipes*, Temminck and Schlegel, 1850), zebrafish (*Danio rerio*, Hamilton, 1822), Japanese pufferfish (*Takifugu rubripes*, Temminck and Schlegel, 1850), and chicken (*Gallus gallus*, Linnaeus, 1758) (30:70, female: male; XX/XY sex-determination type)

Locus ID	Quality of the sequencing data			Chromosomal locations			
	Call Rate	AvgReadDepth	RepAvg	*Oryzias latipes*	*Danio rerio*	*Takifugu rubripes*	*Gallus gallus*
PA57951923	0.94	35.35	-	13	24	-	-
PA57952305	0.94	38.13	-	-	17	-	-
PA57952528	0.94	14.04	-	-	8	-	-
PA57954112	0.94	5.09	-	-	-	-	-
PA57954346	0.88	48.88	-	-	-	-	-
PA57955497	0.88	12.46	-	-	-	-	-
PA57965434	0.81	15.58	-	1	20	15	-
PA57970101	0.88	76.00	-	-	-	-	-
PA57973663	0.94	78.71	-	-	23	13	-
PA57951108	0.97	12.50	-	-	24		Z
PA57951918	0.97	10.38	-	-			-
PA57952399	0.97	19.23	-	2	7	1	-
PA57952700	0.97	46.79	-	-	-	-	-
PA57953678	0.97	9.65	-	-	-	-	-
PA57954874	0.97	23.00	-	-	-	-	-
PA57959662	0.97	46.71	-	-	-	-	-
PA57961377	0.97	7.14	-	-	-	-	-
PA57974767	0.97	7.43	-	-	-	-	-
PA57951050	0.91	5.18	-	-	-	-	-
PA57951455	0.91	31.52	-	-	-	-	-
PA57952045	0.91	31.75	-	17	3	-	-
PA57952346	0.91	6.33	-	-	2	19	-
PA57952678	0.91	6.86	-	-	-	-	-
PA57953243	0.91	20.77	-	-	25	-	-

Locus ID	Quality of the sequencing data			Chromosomal locations			
	Call Rate	AvgReadDepth	RepAvg	*Oryzias latipes*	*Danio rerio*	*Takifugu rubripes*	*Gallus gallus*
PA57953610	0.91	12.83	-	-	18	1	-
PA57969727	0.91	26.20	-	-	-	-	-
PA57973735	0.91	6.46	-	-	-	-	-
PA57975512	0.91	7.22	-	10	1,5,23	-	-
PA57952865	0.84	9.30	-	-	-	-	-
PA57951768	0.94	49.61	-	-	-	-	-
PA57954926	0.94	46.92	-	-	-	-	-
PA57969006	0.94	10.29	-	-	-	-	-
PA57951177	0.88	14.67	-	17	20	-	-
PA57953141	0.88	6.39	-	-	7	-	-
PA57975127	0.81	20.37	-	16	18	7	-
PA57951819	0.97	24.86	-	-	-	-	-
PA57952390	0.97	8.62	-	-	-	-	-
PA57958703	0.97	19.36	-	-	-	12	-
PA57966898	0.97	5.00	-	16	6	-	-
PA57972656	0.97	5.59	-	-		-	-
PA57951125	0.91	24.23	-	-	12	-	-
PA57954990	0.91	6.04	-	-		-	-
PA57951104	0.94	19.08	-	-	19	-	-
PA57965520	0.94	54.63	-	-	-	-	-
PA57950959	0.88	8.41	-	-	-	-	-
PA57974251	0.81	84.54	-	-	-	-	-
PA57953265	0.91	20.89	-	-	9	-	-
PA57953632	0.97	17.42	-	-	-	-	-
SNP57967051	1.00	-	1.00	-	13	-	-
SNP57962593	1.00	-	1.00	-	-	-	-
SNP57972449	1.00	-	1.00	-	-	-	-
SNP57963140	1.00	-	1.00	-	-	-	-
SNP57963962	1.00	-	1.00	-	-	-	-
SNP57967509	1.00	-	1.00	-	-	-	-
SNP57968632	1.00	-	1.00	-	17	-	-
SNP57961629	1.00	-	1.00	-	9	-	-
SNP57960302	1.00	-	1.00	-	9	-	-
SNP57973211	1.00	-	1.00	-	-	-	-
SNP57959934	0.97	-	1.00	-	-	-	-

Locus ID	Quality of the sequencing data			Chromosomal locations			
	Call Rate	AvgReadDepth	RepAvg	*Oryzias latipes*	*Danio rerio*	*Takifugu rubripes*	*Gallus gallus*
SNP57962315	0.97	-	1.00	-	-	-	-
SNP57970848	0.97	-	1.00	-	-	-	-
SNP57973026	0.97	-	1.00	-	-	-	-
SNP57974213	0.97	-	0.94	-	-	-	-
