# Supplementary material for: Genome-wide SNP analysis provides insights into the XX/XY sex-determination system in silver barb (Barbonymus gonionotus)
Source: Genomics Inform. 2023 Dec 29;21(4):e47. doi: 10.5808/gi.23075 (PMC10788355; doi:10.5808/gi.23075)
Supplement: Supplementary Fig. 1. — In silico chromosome mapping showing the distribution of sex-linked loci of the silver barb to the chromosome-level assembly of the common barbel. [file gi-23075-Supplementary-Fig-1.pdf]

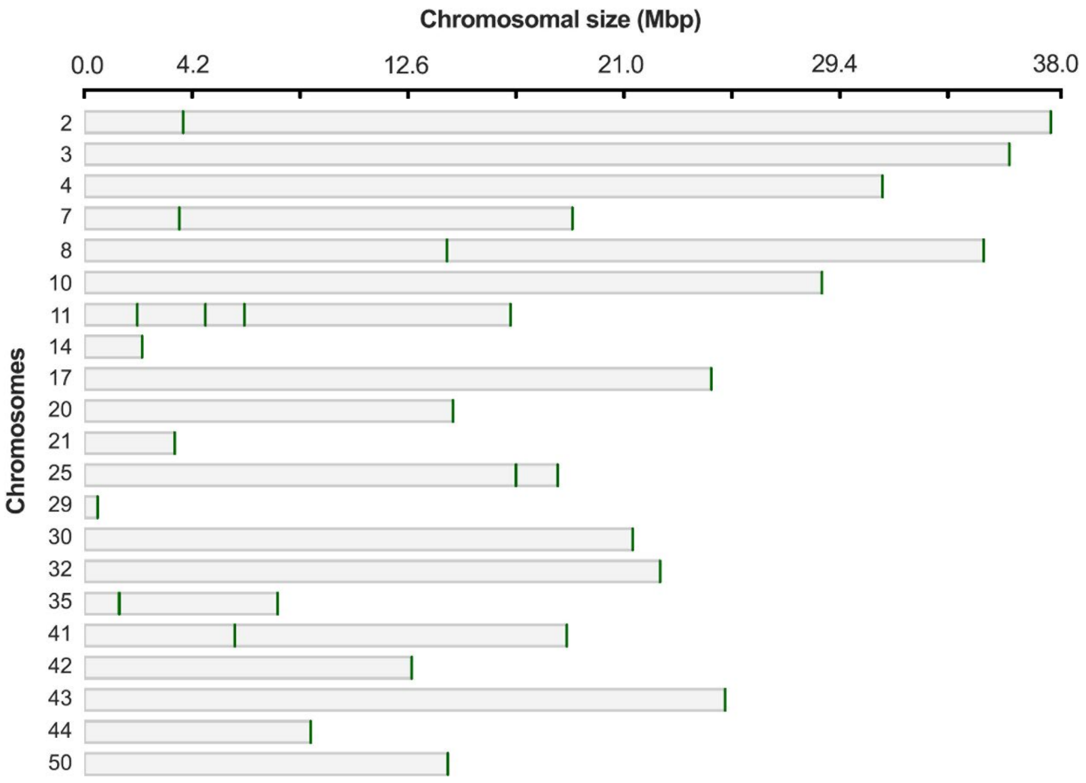

Supplementary Fig. 1. In silico chromosome mapping showing the distribution of sex-linked loci of the silver barb to the chromosome-level assembly of the common barbel.
