# Supplementary material for: Genome-wide SNP analysis provides insights into the XX/XY sex-determination system in silver barb (Barbonymus gonionotus)
Source: Genomics Inform. 2023 Dec 29;21(4):e47. doi: 10.5808/gi.23075 (PMC10788355; doi:10.5808/gi.23075)
Supplement: Supplementary Fig. 2. — Gene ontology (GO) functional classification of specific loci of the silver barb using Blast2GO. Histograms of the frequency of transcripts annotated to specific GO categories, viz., biological process, molecular functions, and cellular components, are represented using orange, blue, and green bars, respectively. [file gi-23075-Supplementary-Fig-2.pdf]

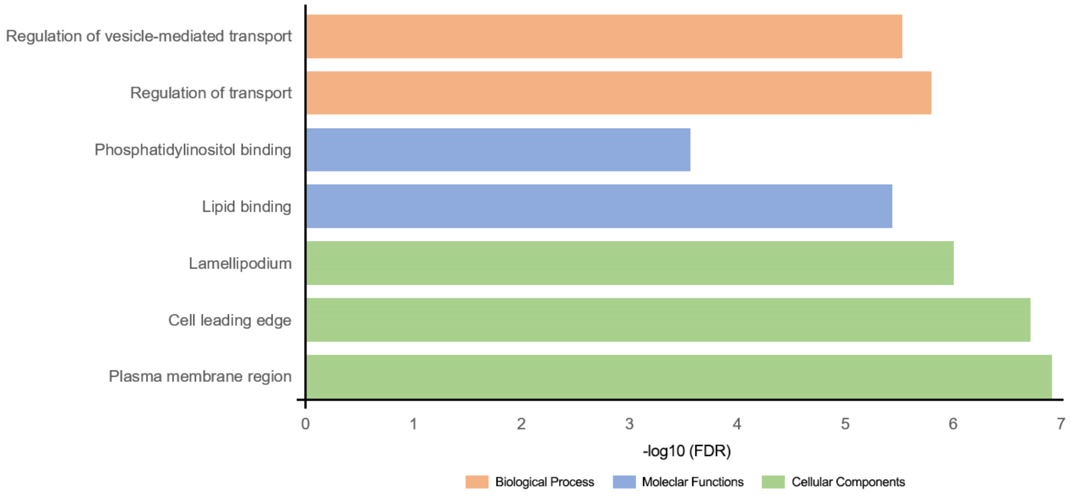

Supplementary Fig. 2. Gene ontology (GO) functional classification of specific loci of the silver barb using Blast2GO. Histograms of the frequency of transcripts annotated to specific GO categories, viz., biological process, molecular functions, and cellular components, are represented using orange, blue, and green bars, respectively.
